# SFMBT2 (Scm-like with four mbt domains 2) negatively regulates cell migration and invasion in prostate cancer cells

**DOI:** 10.18632/oncotarget.10198

**Published:** 2016-06-21

**Authors:** Jungsug Gwak, Jee Yoon Shin, Kwanghyun Lee, Soon Ki Hong, Sangtaek Oh, Sung-Ho Goh, Won Sun Kim, Bong Gun Ju

**Affiliations:** ^1^ Department of Life Science, Sogang University, Seoul 121-742, Republic of Korea; ^2^ Department of Bio and Fermentation Convergence Technology, Kookmin University, Seoul 136-702, Republic of Korea; ^3^ Research Institute, National Cancer Center, Goyang, Gyeonggi-do 410-769, Republic of Korea

**Keywords:** prostate cancer, metastasis, SFMBT2, gene regulation

## Abstract

Metastatic prostate cancer is the leading cause of morbidity and mortality in men. In this study, we found that expression level of SFMBT2 is altered during prostate cancer progression and has been associated with the migration and invasion of prostate cancer cells. The expression level of SFMBT2 is high in poorly metastatic prostate cancer cells compared to highly metastatic prostate cancer cells. We also found that SFMBT2 knockdown elevates MMP-2, MMP-3, MMP-9, and MMP-26 expression, leading to increased cell migration and invasion in LNCaP and VCaP cells. SFMBT2 interacts with YY1, RNF2, N-CoR and HDAC1/3, as well as repressive histone marks such as H3K9me2, H4K20me2, and H2AK119Ub which are associated with transcriptional repression. In addition, SFMBT2 knockdown decreased *KAI1* gene expression through up-regulation of *N-CoR* gene expression. Expression of SFMBT2 in prostate cancer was strongly associated with clinicopathological features. Patients having higher Gleason score (≥ 8) had substantially lower SFMBT2 expression than patients with lower Gleason score. Moreover, tail vein or intraprostatic injection of SFMBT2 knockdown LNCaP cells induced metastasis. Taken together, our findings suggest that regulation of SFMBT2 may provide a new therapeutic strategy to control prostate cancer metastasis as well as being a potential biomarker of metastatic prostate cancer.

## INTRODUCTION

The number of newly diagnosed prostate cancer patients has decreased slightly, but newly diagnosed patients in the United States is more than 200,000 cases per year [1]. The prostate-specific antigen (PSA) is the most widely used for early detection of prostate cancer, risk classification, and monitoring of the disease although it is not specific for prostate cancer [2, 3]. Therefore, many efforts have been made to identify new biomarkers to increase accuracy of prostate cancer diagnosis, and recently new biomarkers such as SRPK1, CXCL12, and TMPRSS4 have been reported [4–6].

It has been reported that 4% of prostate cancer patients in the United State develop a distant metastasis, but the 5-year survival rate is only 28% compared to 100% in localized and regional disease [7]. Prostate cancer cells are able to infiltrate into the lymphatic system or blood stream, and spread mainly to the liver, lung, lymph nodes and bone [8]. Abnormal control of TGF signaling is known to play an important role in promoting tumor metastasis [9]. Up-regulation of the Akt/mTOR pathway by inactivation of PTEN is also associated with invasion and metastasis of prostate cancer, which has led to development of drugs to inhibit this pathway [10, 11]. In addition, increased expression of matrix metalloproteinases (MMPs) has been shown to be associated with prostate cancer progression and metastasis [11, 12].

Polycomb group (PcG) proteins regulate the expression of developmental genes, and abnormal control of the PcG proteins is known to cause cancer [13, 14]. For example, histone H3K27 methyltransferase EZH2 is upregulated in various types of cancer such as cancer of the breast, colon and prostate [15]. Moreover, EZH2 has received attention as a target for cancer treatment because EZH2-mediated tri-methylation of histone H3K27 results in inactivation of tumor suppressor genes such as *PSP94* and *p16^INK4a^* [16–18]. Overexpression of the YY1 has been reported in various cancers including that of breast and prostate [19, 20]. YY1 negatively regulates p53 through proteasome-dependent ubiquitination [21]. YY1 also interacts with cell cycle regulators such as cyclin D, c-Myc and Rb, resulting in abnormal cell proliferation [22].

Recently, SFMBT2, another PcG protein [23], was shown to be involved in prostate cancer cell growth. SFMBT2 interacts with YY1 and regulates cell growth through repression of the *HOXB13* gene in DU145 prostate cancer cells [24]. SFMBT has an MBT (malignant brain tumor) domain, which is important for gene regulation by recognizing and binding to methylated lysine residue of histone H3 and H4 tails [25]. In fact, MBT domains of *Drosophila* SFMBT preferentially bind to mono- and di-methylated histone H3K9 and H4K20 peptides, which are associated with transcriptional repression [23, 26]. Human SFMBT2 also binds to methylated lysine residue of histone H3 and H4, which are found in inactive genes, indicating that SFMBT2 may be involved in recognizing repressive hypermethylated histones and maintaining inactive chromatin. Similarly, SFMBT1 forms a complex with LSD1 and CoREST. This complex further induces inactive chromatin and transcriptional repression of replication-dependent histone genes [27].

In this study, we investigated the role of SFMBT2 in metastasis of prostate cancer. Knockdown of SFMBT2 increases prostate cancer cell migration and invasion via direct repression of target genes such as *MMP-9*, *MMP-26*, and *N-CoR* in LNCaP and VCaP cells. In addition, a metastasis suppressor *KAI1* gene is regulated indirectly by SFMBT2. Interestingly, expression level of SFMBT2 inversely correlates with Gleason score in prostate cancer patients. Moreover, we found that tail vein or intraprostatic injection of SFMBT2 knockdown LNCaP cells significantly induces metastasis, indicating that SFMBT2 acts as a metastasis suppressor in prostate cancer *in vivo*.

## RESULTS

### SFMBT2 regulates cell migration and invasion in LNCaP cells

**Figure 1:**
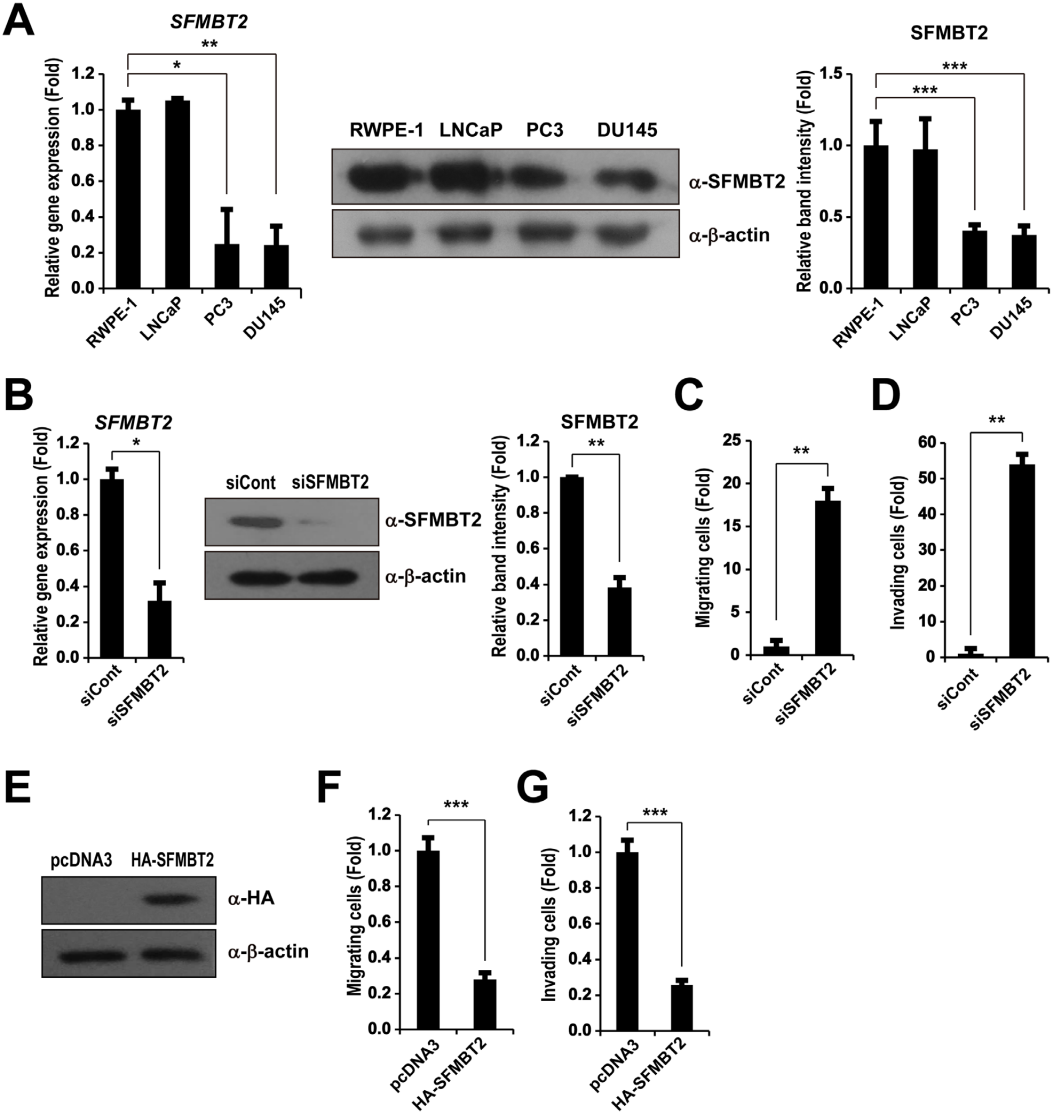
SFMBT2-mediated cell migration and invasion in LNCaP cells **A.** Differential expression level of SFMBT2 in normal prostate and prostate cancer cell lines. Transcripts of *SFMBT2* and *GAPDH* were determined by quantitative PCR in RWPE-1, LNCaP, PC3, and DU145 cells (n=3). The cell lysates were immunoblotted with anti-SFMBT2 and anti-β-actin antibodies, respectively (n=3). Western blots were analyzed quantitatively. **B.** Knockdown of SFMBT2 results in increased cell migration and invasion in LNCaP cells. After control (siCont) or SFMBT2 siRNA (siSFMBT2) were transfected, LNCaP cells were subjected to RNA and protein extraction (n=3). Transcripts of *SFMBT2* and *GAPDH* were determined by quantitative PCR. The cell lysates were immunoblotted with anti-SFMBT2 and anti-β-actin antibodies, respectively. Western blots were analyzed quantitatively. **C.** After control or SFMBT2 siRNA were transfected, LNCaP cells were subjected to a cell migration assay using a modified Boyden chamber containing uncoated Transwell polycarbonate membrane filters (n=3). The migrated cells stained with cresyl violet were counted. **D.** After control or SFMBT2 siRNA were transfected, LNCaP cells were subjected to a cell invasion assay using a Biocoat Matrigel invasion chambers (n=3). Invading cells on the membrane stained with cresyl violet were counted. **E.** PC3 cells were transfected with pcDNA3 or pcDNA3-SFMBT2-HA plasmid (n=3). The cell lysates were immunoblotted with anti-HA and anti-β-actin antibodies, respectively. **F, G.** After PC3 cells were transfected with pcDNA3 or pcDNA3-SFMBT2-HA plasmid, cell migration assay (n=3) and invasion assay (n=3) were performed. All data represent mean ± S.E.M. Significance values were * *P*≤0.05, ** *P*≤0.01 and *** *P*≤0.005.

We found previously that mammalian SFMBT2, a polycomb gene (PcG), regulates cell growth in prostate cancer cells [24]. To further evaluate a possible role of SFMBT2 in prostate cancer progression, we first analyzed expression level of SFMBT2 in various prostate cancer cells lines. The expression level of SFMBT2 was high in normal RWPE-1 prostate cells and poorly metastatic LNCaP prostate cancer cells, but low in highly metastatic prostate cancer cells, such as PC3 and DU145, indicating that SFMBT2 expression is likely related to metastasis (Figure 1A).

We next decided to investigate whether knockdown of SFMBT2 by siRNA affects the cell migration and invasion, which are main features of metastasis, using poorly metastatic LNCaP cells [28, 29]. Quantitative PCR and Western blotting showed SFMBT2 siRNA efficiently down-regulated SFMBT2 expression in LNCaP cells (Figure 1B). When SFMBT2 knockdown LNCaP cells were subjected to cell migration assay using a modified Boyden chamber, we found increased cell migration as compared to control siRNA-transfected cells (Figure 1C). In addition, knockdown of SFMBT2 further resulted in increased cell invasion using a Biocoat Matrigel invasion chamber (Figure 1D). Consistently, we also found that overexpression of SFMBT2 decreases cell migration and invasion in highly metastatic PC3 cells, which expresses low level of SFMBT2 (Figure 1A and 1E-1G). These results suggest that SFMBT2 may function as a negative regulator in cell migration and invasion in prostate cancer cells.

### Knockdown of SFMBT2 increases expression and activity of MMP genes

**Figure 2:**
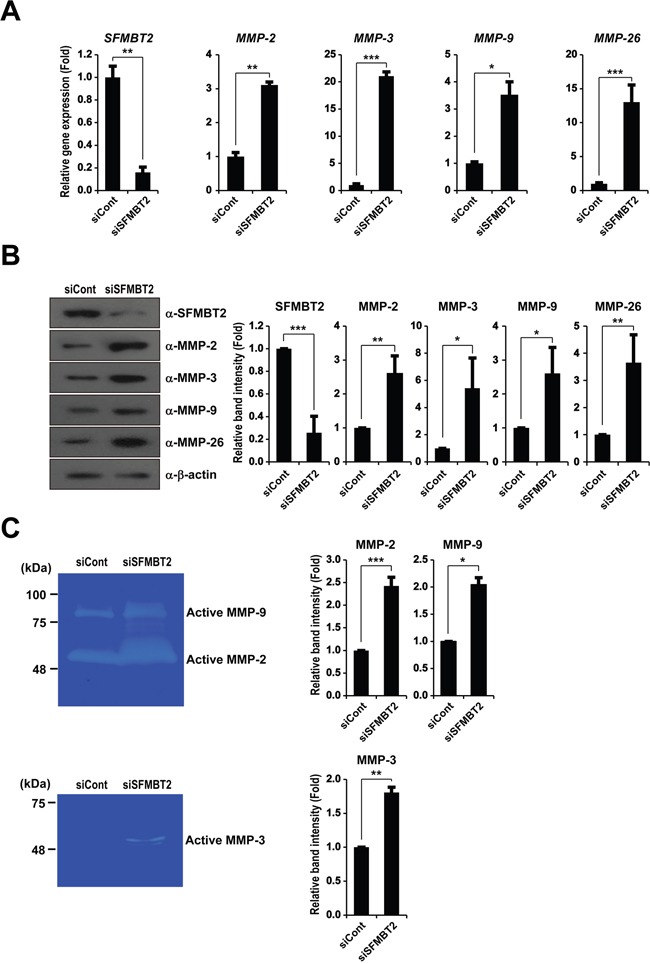
SFMBT2 regulates expression of matrix metalloproteinase in LNCaP cells Knockdown of SFMBT2 increases expression of the *MMP-2*, *MMP-3*, *MMP-9*, and *MMP-26* gene in LNCaP cells. **A.** After control or SFMBT2 siRNA were transfected, LNCaP cells were subjected to RNA extraction (n=3). Transcripts of *MMP-2*, *MMP-3*, *MMP-9*, *MMP-26*, and *GAPDH* were determined by quantitative PCR. **B.** The cell lysates were immunoblotted with anti-MMP-2, anti-MMP-3, anti-MMP-9, and anti-MMP-26 antibodies, respectively (n=3). Anti-β-actin was used as a loading control. Western blots were analyzed quantitatively. **C.** Elevation of enzyme activities of MMP-2, MMP-3, and MMP-9 in SFMBT2 knockdown LNCaP cells. Zymography was performed using the lysates from control and SFMBT2 siRNA-transfected cells (n=3). Equal amounts of the lysate were used for each reaction. Zymography was analyzed quantitatively. All data represent mean ± S.E.M. Significance values were * *P*≤0.05, ** *P*≤0.01, and *** *P*≤0.005.

Given that SFMBT2 participates in transcriptional repression, and MMPs are critical for cell migration and invasion through proteolysis of the extracellular matrix [24, 30, 31], we tested whether SFMBT2 regulates MMP gene expression. We examined expression of MMP genes including *MMP-2*, *MMP-3*, *MMP-7*, *MMP-9*, *MMP-13*, *MMP-14*, *MMP-15*, and *MMP-26* that are known to be up-regulated during prostate cancer progression [11]. Among MMPs, we found a significantly increased expression of the *MMP-2*, *MMP-3*, *MMP-9*, and *MMP-26* genes in SFMBT2 knockdown LNCaP cells (Figure 2A and Supplementary Figure S1). We also performed experiments using other androgen-dependent prostate cancer VCaP cells [32, 33]. Consistent with the results from LNCaP cells, knockdown of SFMBT2 resulted in increased expression of *MMP-2*, *MMP-3*, *MMP-9* and *MMP-26* genes as well as increases cell migration and invasion in VCaP cells (Supplementary Figure S2).

We further confirmed the up-regulation of MMP expression by Western blot analysis as shown in Figure 2B. Zymography consistently revealed elevated enzyme activity of MMP-2, MMP-9, and MMP-3 in SFMBT2 knockdown LNCaP cells (Figure 2C). Collectively, these results suggest that SFMBT2 may act as a transcriptional repressor of MMP genes to prevent cell migration and invasion in LNCaP and VCaP cells.

### Recruitment of SFMBT2, YY1, RNF2, N-CoR, and HDAC1/3 to MMP-9 and MMP-26 gene promoters

**Figure 3:**
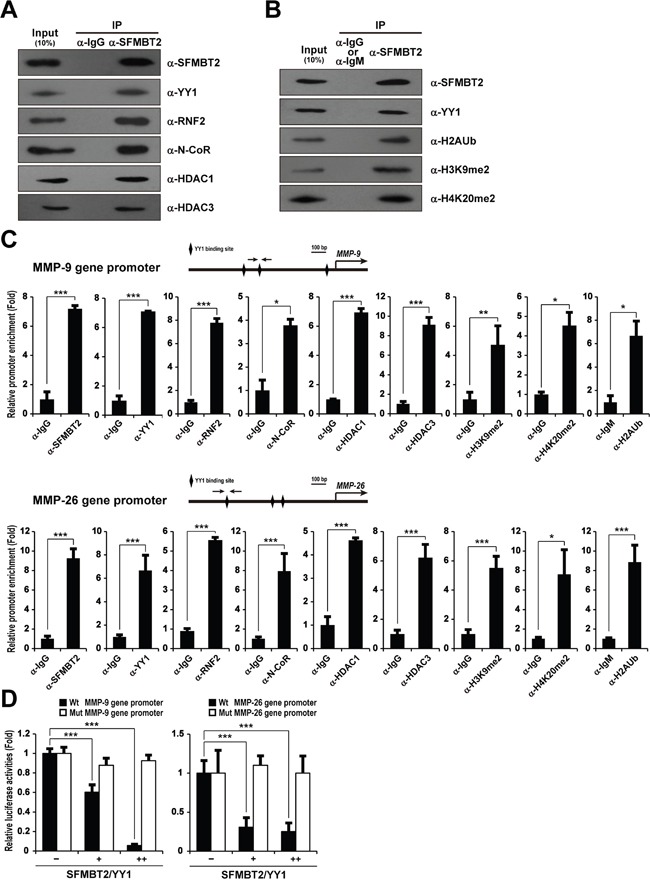
Recruitment of SFMBT2, YY1, RNF2, N-CoR, HDAC1, and HDAC3 to the *MMP-9* and *MMP-26* gene promoters in LNCaP cells **A, B.** SFMBT2 interacts with YY1, RNF2, N-CoR, HDAC1, HDAC3, and repressive histone marks. The lysates from LNCaP cells were immunoprecipitated with the anti-SFMBT2 antibody, then immunoblotted with anti-SFMBT2, anti-YY1, anti-RNF2, anti-N-CoR, anti-HDAC1, anti-HDAC3, anti-dimethyl H3K9 (H3K9me2), anti-dimethyl H4K20 (H4K20me2), and anti-mono-ubiquitinated H2AK119 (H2AK119Ub) antibodies, respectively (n=3). Immunoprecipitation with normal IgG or IgM was used as a negative control. **C.** Enrichment of SFMBT2, YY1, RNF2, N-CoR, HDAC1, and HDAC3 on the *MMP-9* and *MMP-26* gene promoters in LNCaP cells. A ChIP assay was performed using anti-SFMBT2, anti-YY1, anti-RNF2, anti-N-CoR, anti-HDAC1, anti-HDAC3, anti-H3K9me2, anti-H4K20me2, and anti-H2AK119Ub antibodies, respectively (n=3). The occupancy of each protein was determined by quantitative PCR in *MMP-9* and *MMP-26* gene promoters encompassing the YY1 binding site using oligonucleotide primers (arrows). ChIP using normal IgG or IgM was performed as a negative control. **D.** Repression of the *MMP-9* and *MMP-26* gene promoter reporter activity by over-expression of SFMBT2 and YY1. However, over-expression of SFMBT2 and YY1 does not affect the reporter activity of *MMP-9* and *MMP-26* gene promoters containing a mutated the YY1 binding site. LNCaP cells were transiently transfected with the human *MMP-9* and *MMP-26* gene promoter-driven firefly luciferase reporter vector in conjunction with a control *Renilla* luciferase expression vector (n=3). Expression vectors for SFMBT2 and YY1 were transfected. Reporter activity is represented as fold activation relative to *Renilla* luciferase activity. All data represent mean ± S.E.M. Significance values were * *P*≤0.05, ** *P*≤0.01, and *** *P*≤0.005.

In order to study the molecular mechanism underlying SFMBT2-mediated transcriptional repression of MMP genes, we first investigated the interaction of SFMBT2 with YY1 and other cofactors including co-repressors. Since SFMBT2 is found to interact with YY1, which is a mammalian ortholog of *Drosophila* PHO [24, 34, 35], we further confirmed interaction of SFMBT2 with YY1 in LNCaP cells (Figure 3A). Consistent with a previous report [27], we also found that SFMBT2 interacts with RING1B/RNF2 E3 ubiquitin ligase, which mediates mono-ubiquitination of H2AK119, in LNCaP cells. SFMBT2 interaction with transcriptional co-repressor N-CoR, HDAC1, and HDAC3 were clearly demonstrated (Figure 3A). The MBT domain of the SFMBT family proteins preferentially binds to methylated histone H3K9 and H4K20 peptides [23–26]. We also confirmed interaction of SFMBT2 with repressive histone marks such as di-methylated H3K9 (H3K9me2) and di-methylated H4K20 (H4K20me2) (Figure 3B). Moreover, SFMBT2 was discovered to be associated with mono-ubiquitinated histone H2AK119 (H2AUb), which is found frequently in silenced genes [36], indicating that SFMBT2 may play a role in maintenance of the inactive state of the MMP genes though an association with repressive histone marks in LNCaP cells.

To investigate recruitment of SFMBT2, YY1, RNF2, N-CoR, HDAC1, and HDAC3 to MMP gene promoters, we first identified putative YY1 binding sites on each genes using *in silico* bioinformatic analysis (Supplementary Figure S3). We further confirmed YY1 binding site using a series of ChIP assay and mutagenesis. After immunoprecipitation with each of the antibodies, quantitative PCR was performed to amplify the MMP genes' promoter region containing the YY1 binding sites. We found that SFMBT2, YY1, RNF2, N-CoR, HDAC1, and HDAC3 are bound significantly to the *MMP-9* and *MMP-26* gene promoters (Figure 3C). However, we did not find a significant recruitment of SFMBT2 and YY1 on the *MMP-2* and *MMP-3* gene promoters, indicating that SFMBT2 may regulate *MMP-2* and *MMP-3* indirectly (Supplementary Figure S4). H3K9me2 and H4K20me2 were significantly enriched at the *MMP-9* and *MMP-26* gene promoters. Consistent with recruitment of RNF2, a significant enrichment of H2AK119Ub was also observed at the *MMP-9* and *MMP-26* gene promoters (Figure 3C). In contrast, we failed to detect a significant enrichment of SFMBT2, YY1, RNF2, N-CoR, HDAC1, and HDAC3 as well as repressive histone marks at the gene promoter region of *GAPDH*, which is expressed constitutively in LNCaP cells (Supplementary Figure S5). Similarly, when SFMBT2 and YY1 were over-expressed in LNCaP cells carrying *MMP-9* and *MMP-26* gene promoter reporters, a significant decrease in report activity was observed (Figure 3D). However, we did not observe a significant decreased reporter activity in *MMP-9* and *MMP-26* gene promoter reporters containing a mutated YY1 binding site (Figure 3D).

### SFMBT2 up-regulates *KAI1* gene expression through down-regulation of N-CoR

**Figure 4:**
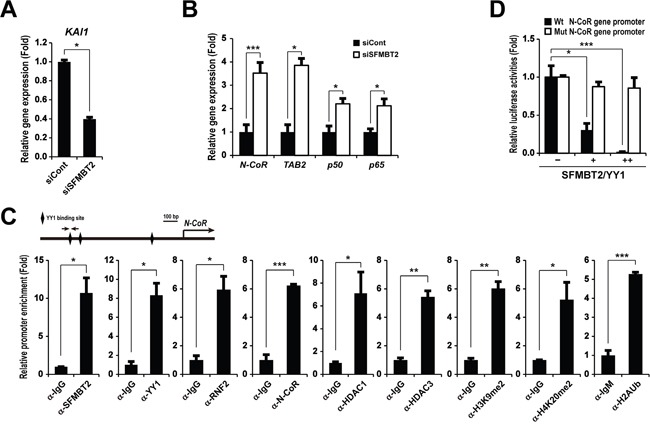
SFMBT2 indirectly regulates expression of *KAI1* metastasis suppressor gene through repression of *N-CoR* gene expression in LNCaP cells **A.** Down-regulation of *KAI1* gene expression in SFMBT2 knockdown LNCaP cells. Transcripts of *KAI1* and *GAPDH* were determined by quantitative PCR (n=3). **B.** Up-regulation of transcriptional repressors for *KAI1* gene in SFMBT2 knockdown LNCaP cells. Transcripts of *N-CoR*, *TAB2*, *p50*, *p65*, and *GAPDH* were determined by quantitative PCR (n=3). **C.** Enrichment of SFMBT2, YY1, RNF2, N-CoR, HDAC1, HDAC3, di-methyl H3K9, di-methyl H4K20, and mono-ubiquitinated H2AK119 on the *N-CoR* gene promoter in LNCaP cells. A ChIP assay was performed in LNCaP cells using anti-SFMBT2, anti-YY1, anti-RNF2, anti-N-CoR, anti-HDAC1, anti-HDAC3, anti-H3K9me2, anti-H4K20me2, and anti-H2AK119Ub antibodies, respectively (n=3). The occupancy of each protein was determined by quantitative PCR in the *N-CoR* gene promoter encompassing the YY1 binding site using oligonucleotide primers (arrows). ChIP using normal IgG or IgM was performed as a negative control. **D.** Repression of *N-CoR* gene promoter reporter activity by over-expression of SFMBT2 and YY1. However, over-expression of SFMBT2 and YY1 does not affect reporter activity of the *N-CoR* gene promoter containing mutated the YY1 binding site. LNCaP cells were transiently transfected with the human *N-CoR* gene promoter-driven firefly luciferase reporter vector in conjunction with a control *Renilla* luciferase expression vector (n=3). Expression vectors for SFMBT2 and YY1 were transfected in combination. Reporter activity is represented as fold activation relative to *Renilla* luciferase activity. All data represent mean ± S.E.M. Significance values were * *P*≤0.05, ** *P*≤0.01, and *** *P*≤0.005.

Since the low expression level of the KAI1 has been shown to relate to prostate metastasis [37, 38], we tested whether SFMBT2 regulates *KAI1* gene expression. Interestingly, expression of the *KAI1* gene was down-regulated in SFMBT2 knockdown LNCaP cells (Figure 4A). Thus, we tested the possibility that SFMBT2 may negatively regulate transcriptional repressors for the *KAI1* gene. Among known transcriptional repressors for the *KAI1* gene [39], we found up-regulation of *N-CoR*, *TAB2*, and *NF-κB* (*p65* and *p50*) in SFMBT2 knockdown LNCaP cells (Figure 4B). To investigate whether SFMBT2/YY1 directly represses *N-CoR*, *TAB2*, *p65*, and *p50* gene expression, we identified functional YY1 binding sites in each gene using *in silico* bioinformatic analysis (Supplementary Figure S3), ChIP assay, and mutagenesis. Our results indicated that SFMBT2 and YY1 are bound significantly to the *N-CoR* gene promoter, while we did not observe a significant recruitment of SFMBT2 and YY1 on other genes (Figure 4C and Supplementary Figure S6). We also observed a significant enrichment of RNF2, N-CoR, HDAC1, and HDAC3 as well as repressive histone marks on the *N-CoR* gene promoter (Figure 4C). Over-expression of SFMBT2 and YY1 repressed reporter activity of the *N-CoR* gene promoter while reporter activity of the *N-CoR* gene promoter containing a mutated YY1 binding site did not changed by over-expression of SFMBT2 and YY1 (Figure 4D).

### NF-κB up-regulates MMP-9, MMP-26, and N-CoR gene expression in SFMBT2 knockdown LNCaP cells

**Figure 5:**
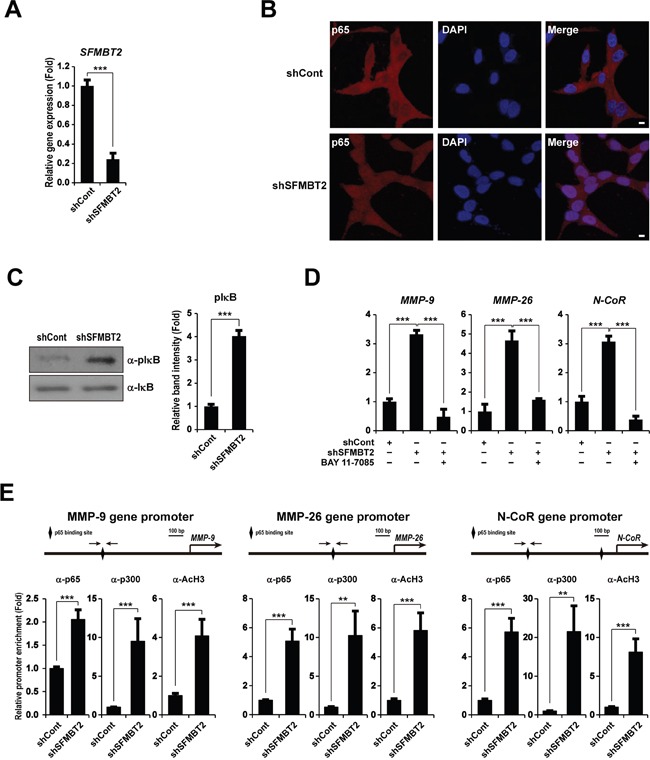
NF-κB activates *MMP-9*, *MMP-26*, and *N-CoR* gene expression in SFMBT2 knockdown LNCaP cells **A.** After control or SFMBT2 shRNA were transfected stably in LNCaP cells, transcripts of *SFMBT2* and *GAPDH* were determined by quantitative PCR (n=3). **B.** NF-κB activation in SFMBT2 knockdown LNCaP cells. Knockdown of SFMBT2 results in the nuclear localization of NF-κB p65. Representative photomicrographs of NF-κB p65 in SFMBT2 knockdown LNCaP cells. After control or SFMBT2 shRNA were transfected stably, cells were immunostained with the anti-NF-κB p65 antibody (n=3). Nuclei were identified using DAPI staining. Scale bar, 10 μm. **C.** Increased phosphorylation of IκB in SFMBT2 knockdown LNCaP cells. After control or SFMBT2 shRNA were transfected stably, lysates were immunoblotted with the anti-IκB and anti-phospho IκB (pIκB) antibodies, respectively (n=3). Western blots were analyzed quantitatively. **D.** Inactivation of NF-κB by BAY 11-7085 attenuates up-regulation of the *MMP-9*, *MMP-26*, and *N-CoR* genes in SFMBT2 knockdown LNCaP cells. After control or SFMBT2 shRNA were transfected stably, cells were treated with DMSO control or BAY 11-7085. Transcripts of *MMP-9*, *MMP-26*, *N-CoR*, and *GAPDH* were determined by quantitative PCR (n=3). **E.** Enrichment of NF-κB p65 and p300 on the *MMP-9*, *MMP-26*, and *N-CoR* gene promoters in SFMBT2 knockdown LNCaP cells. After control or SFMBT2 shRNA were transfected stably, a ChIP assay was performed in LNCaP cells using anti-NF-κB p65, anti-p300, and anti-acetylated H3 antibodies, respectively (n=3). The occupancy of each protein was determined by quantitative PCR in *MMP-9*, *MMP-26*, and *N-CoR* gene promoters encompassing the NF-κB binding site using oligonucleotide primer (arrows). ChIP using normal IgG or IgM was performed as a negative control. All data represent mean ± S.E.M. Significance values were ** *P*≤0.01 and *** *P*≤0.005.

It has been reported frequently that YY1 acts as a transcriptional activator and repressor on the same target gene [40–43]. We thus investigated the possibility that YY1 is involved in *MMP-9*, *MMP-26*, and *N-CoR* gene activation in SFMBT2 knockdown LNCaP cells, which were transfected shSFMBT2 stably (shSFMBT2) (Figure 5A). A ChIP assay revealed little to no recruitment of YY1 to the *MMP-9*, *MMP-26*, and *N-CoR* gene promoters (Supplementary Figure S7), indicating that YY1 is not required for *MMP-9*, *MMP-26*, and *N-CoR* gene activation in SFMBT2 knockdown LNCaP cells. Given that *NF-κB* (*p65* and *p50*) were up-regulated by knockdown of SFMBT2 (Figure 4B), we examined the involvement of NF-κB p65 in *MMP-9*, *MMP-26*, and *N-CoR* gene activation in SFMBT2 knockdown LNCaP cells. As shown in Figure 5B, nuclear localization of p65 was observed in SFMBT2 knockdown LNCaP cells (shSFMBT2) as compared to LNCaP cells transfected with control shRNA stably (shControl). Consistently, knockdown of SFMBT2 resulted in increased phosphorylation of IκB (Figure 5C). We further confirmed our results using BAY 11-7085, an inhibitor of IκBα phosphorylation, to prevent NF-κB activation. BAY 11-7085 treatment attenuated up-regulation of *MMP-9*, *MMP-26*, and *N-CoR* gene expression in SFMBT2 knockdown LNCaP cells (Figure 5D). We also performed ChIP analysis in gene promoters encompassing the NF-κB binding site (Supplementary Figure S3). As expected, recruitment of NF-κB p65 and the p300 co-activator was increased significantly to the *MMP-9*, *MMP-26*, and *N-CoR* gene promoters in SFMBT2 knockdown LNCaP cells as compared to control LNCaP cells (Figure 5E). Enrichment of acetylated H3 to *MMP-9*, *MMP-26*, and *N-CoR* gene promoters was also increased in SFMBT2 knockdown LNCaP cells (Figure 5E).

### SFMBT2 expression in prostate cancer patients

**Figure 6:**
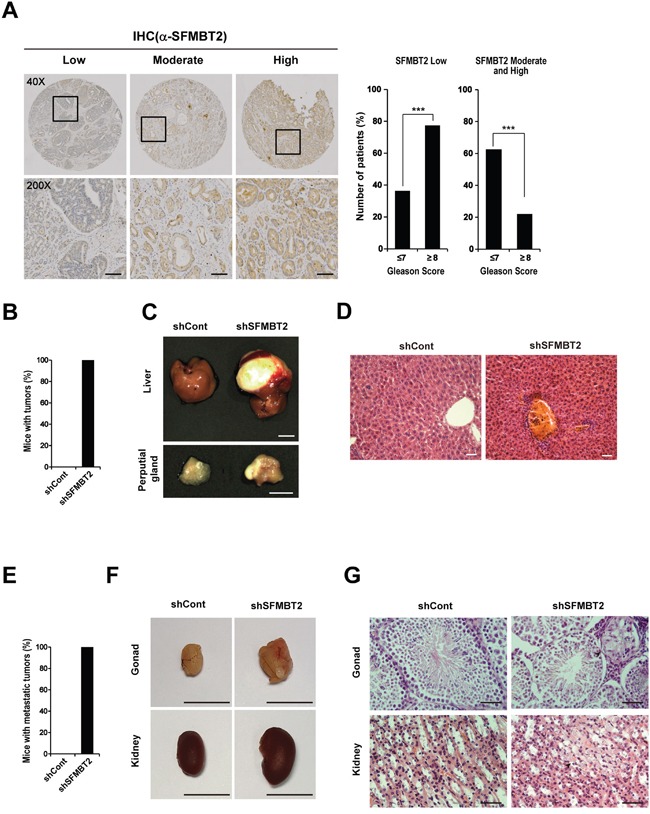
SFMBT2 negatively regulates prostate cancer metastasis *in vivo* **A.** Inverse relationship of SFMBT2 expression with Gleason score in prostate cancer. Expression level of SFMBT2 in the specimens with Gleason scores of ≥ 8 was lower than in those with Gleason scores of ≤ 7. Fisher's exact test was used to calculate P values. Immunohistochemical staining of a tissue array from prostate cancer patients was performed using the anti-SFMBT2 antibody. Representative photomicrographs show the low, moderate, and high expression of SFMBT2. Scale bar, 50 μm. **B.** LNCaP cells (1×10^6^ cells) transfected stably with control (shCont)- or SFMBT2 shRNA (shSFMBT2)-GFP were injected into the tail vein of nude mice, respectively (n=3/group). All mice injected with shSFMBT2-GFP LNCaP cells had tumors. **C.** Representative photomicrographs show liver and perputial gland tumors in shSFMBT2-GFP LNCaP cells-injected mice at week 15 post injection. Scale bar; 1 cm. **D.** Infiltration of shSFMBT2-GFP LNCaP cells in the liver. Livers from control (shCont)- or SFMBT2 shRNA (shSFMBT2)-GFP LNCaP cell-injected mice were fixed, sectioned, and stained with H & E. Scale bar, 50 μm. **E.** LNCaP cells (1×10^6^ cells) transfected stably with control (shCont.)- or SFMBT2 shRNA (shSFMBT2)-GFP were injected into the prostate (dorsal lobe) (n=3/group). All mice injected with shSFMBT2-GFP LNCaP cells had metastatic tumors. **F.** Representative photomicrographs show gonad and kidney tumors in shSFMBT2-GFP LNCaP cells-injected mice at week 5 post injection. Scale bar; 1 cm. **G.** Infiltration of shSFMBT2-GFP LNCaP cells in the gonad and kidney. Gonad and kidney from control (shCont)- or SFMBT2 shRNA (shSFMBT2)-GFP LNCaP cell-injected mice were fixed, sectioned, and stained with H & E. Scale bar, 50 μm. All data represent mean ± S.E.M. Significance values were * *P*≤0.05 and *** *P*≤0.005.

The clinical relevance of SFMBT2 was examined by immunohistochemistry using commercially available prostate cancer tissue arrays (See Materials and Methods). Fifty three samples with various Gleason scores were immunostained using the anti-SFMBT2 antibody (Figure 6A and Supplementary Table S5). In normal prostate tissues, the expression of SFMBT2 was high in 87.5% (7/8 cases) and low in 12.5% (1/8 cases). In contrast, SFMBT2 expression of prostate cancer patient's specimens was low in 60.4 % (32/53 cases), moderate in 32.1% (17/53 cases), and high in 7.5% (4/53 cases) (Supplementary Table S6). Overall, low expression of SFMBT2 appears to be related to prostate cancer. We further analyzed these data to investigate the relationship between expression level of SFMBT2 and Gleason score. We found that 77.42% of analyzed specimens with high Gleason scores of ≥ 8 (24/31 cases) showed low SFMBT2 expression while 22.58% (6+1/31 cases) showed moderate and high SFMBT2 expression (Figure 6A and Supplementary Table S6). In specimens with Gleeson score 4~7, 36.36% (8/22 cases) of specimens showed low SFMBT2 expression while 63.64% (11+3/22 cases) showed moderate and high SFMBT2 expression. These results may suggest that SFMBT2 level inversely correlates with Gleason scores and is related to prognosis of prostate cancer patients such as metastasis and invasion.

### Down-regulation of SFMBT2 induces tumor metastasis *in vivo*

In the following experiments, we investigated whether knockdown of SFMBT2 induces tumor metastasis *in vivo*. We injected stably shSFMBT2-GFP transfected-LNCaP cells, which showed efficient knockdown of SFMBT2, into the tail vein of nude mice (Figure 5A). At 15 weeks after injection, the mice were imaged to detect GFP-positive LNCaP cells. *In vivo* image analysis revealed that a number of GFP-positive cells increased significantly in all of shSFMBT2-GFP LNCaP cell-injected mice as compared with control mice, which were injected with shControl-GFP LNCaP cells (data not shown). All mice injected with shSFMBT2-GFP LNCaP cells had tumors (Figure 6B). Specifically, we observed tumors containing GFP-positive LNCaP cells in the liver and perputial gland of the shSFMBT2-GFP LNCaP cell-injected mice (Figure 6C and Supplementary Figure S8). Histological analysis of the liver tumors showed infiltration of LNCaP cells (Figure 6D). We also performed intraprostatic injection of stably shSFMBT2-GFP transfected-LNCaP cells. Consistently, we found that knockdown of SFMBT2 induces metastasis (Figure 6E). Metastatic tumors of LNCaP cells expressing shSFMBT2-GFP was observed in the gonad and kidney (Figure 6F and 6G), indicating that knockdown of SFMBT2 promotes prostate cancer metastasis.

## DISCUSSION

The mammalian PcG protein SFMBT2 has been shown to play an important role in prostate cancer cell growth through *HOXB13* gene regulation via interaction with YY1 [24]. In this study, we further characterized the function of SFMBT2 in prostate cancer metastasis. We showed that the expression level of SFMBT2 is critical for cell migration and invasion, which are fundamental features of prostate cancer malignancy through direct or indirect regulation of *MMP-2*, *MMP-3*, *MMP-9*, *MMP-26*, *N-CoR*, and *KAI1* gene expression.

It is well known that extracellular matrix (ECM) degradation by MMPs is required for cancer cell migration and invasion [11, 44]. Specifically, increased expression of MMP-2 and MMP-9 has been shown to be associated with prostate cancer progression and metastasis [12, 45]. Other studies also consistently show that inhibition of MMP-2 and MMP-9 expression suppresses the metastatic potential of prostate cancer cells [46, 47]. Although information about MMP-3 is limited regarding prostate cancer progression, it is known that expression of MMP-3 increases in highly metastatic PC3 cells as compared to poorly metastatic LNCaP cells [48]. Invasiveness of PC3 or DU145 cells also seems to be correlated with up-regulation of MMPs including MMP-3 [49–51]. MMP-26 has been shown to activate pro-MMP-9, thereby promoting invasion of prostate cancer cells [52, 53].

Knockdown of SFMBT2 further reveals the dysregulation of the *KAI1* metastasis suppressor gene [37, 38]. The expression level of *KAI1* is significantly low in invasive prostate tumor as compared to prostate intraepithelial neoplasia (GEO data set: GDS2443). Since SFMBT2 functions as a negative transcriptional regulator, we assumed that SFMBT2 may repress the transcriptional repressor(s) for the *KAI1* gene. Among the known transcriptional repressors [39], we found that the *N-CoR* is repressed by SFMBT2 and YY1 directly. In addition, our results indicate that *N-CoR* gene expression is regulated by negative feedback, since N-CoR represses its own transcription. Transcriptional repression by SFMBT2 is achieved by interaction with YY1, RNF2, N-CoR, and HDACs at the *MMP-9*, *MMP-26*, and *N-CoR* gene promoters. In fact, YY1 is implicated in prostate cancer development and progression through its regulation of *PSA* gene expression [54, 55]. We found that E3 ubiquitin ligase RNF2, a PcG protein, is also associated with SFMBT2/YY1 at the promoters. Although the function of RNF2 has not been determined in prostate cancer progression, knockdown of RNF2 by siRNA abolished SNAIL-mediated *E-cadherin* repression and induced cell migration in pancreatic cancer cells [56].

We assumed that YY1 is also required for up-regulation of *MMP-9*, *MMP-26*, and *N-CoR* gene expression in SFMBT2 knockdown LNCaP cells, since YY1 functions as both a transcriptional activator and a repressor for the same target gene [40–43]. However, ChIP analysis clearly indicated the absence of YY1 but the occupancy of NF-κB on the *MMP-9*, *MMP-26*, and *N-CoR* gene promoters, leading to *MMP-9*, *MMP-26*, and *N-CoR* gene activation in SFMBT2 knockdown LNCaP cells. Our study also shows that NF-κB activation in SFMBT2 knockdown LNCaP cells (Figure 5). Previous studies have consistently demonstrated the requirement of NF-κB for up-regulation of the human *MMP-9* gene [57–59]. Although there has been no report thus far on the existence of a functional NF-κB binding site within human *MMP-26* and *N-CoR* gene promoters, we found a putative NF-κB binding site using a promoter reporter and ChIP assays.

Although we do not know how SFMBT2 knockdown results in NF-κB activation at present, recent studies have demonstrated that NF-κB activation maybe associated with metastasis of prostate cancer. NF-κB is activated in highly metastatic prostate cancer cells, such as PC3 and DU145, as compared to poorly metastatic LNCaP cells [59–62]. The nuclear localization of NF-κB was significantly increased in metastatic prostate cancer [60]. Several mechanisms for NF-κB activation have been proposed. For example, LNCaP cells secrete a low level of cytokines, which can activate NF-κB [63, 64]. In addition, genetic alterations such as mutation, amplification, overexpression, and rearrangement of genes involving NF-κB and its signaling also may account for abnormal NF-κB activation in SFMBT2 knockdown LNCaP cells [65–67]. It has been demonstrated that heavy phosphorylation of the IkBα inhibitor by IKK occurs in PC3 and DU145 cells as compared to LNCaP cells [62]. Similarly, we found increased nuclear translocation of NF-κB and phosphorylation of IkBα in SFMBT2 knockdown LNCaP cells (Figure 5A).

The biological significance of SFMBT2 was further confirmed by *in vivo* metastasis experiments. The tail vein or intraprostatic injection of SFMBT2 knockdown LNCaP cells in mice significantly induces metastasis, indicating that SFMBT2 may inhibit metastasis of prostate cancer *in vivo*. In addition, expression level of SFMBT2 in prostate cancer patients is negatively correlated with a high Gleason score (≥ 8), which seems closely related to prostate cancer invasion and metastasis [68–71]. Since the follow-up period of the analyzed specimens in this study is an average 28.3 months, we could not test whether expression level of SFMBT2 relates to survival rate. However, it has been found that SFMBT2 levels positively correlate with survival rate in various types of cancer such as breast (GEO accession; GSE21653, P value; 0.0785), colon (GEO accession; GSE17538, P value; 0.068), and astrocytic gliomas (GEO accession; GSE18166, P value; 0.0788). We believe that further studies with larger number of patients are required to support our results.

In conclusion, our study provides evidence that SFMBT2 regulates prostate cancer metastasis either by direct repression of YY1 target genes such as *MMP-9*, *MMP-26*, and *N-CoR* and by indirect regulation of *MMP-2*, *MMP-3*, and *KAI1* gene expression. Therefore, regulation of SFMBT2 expression or SFMBT2 activity may provide a new therapeutic strategy to suppress cancer cell migration and invasion as well as a potential biomarker in prostate cancer progression.

## MATERIALS AND METHODS

### RNA interference

LNCaP and VCaP cells were transfected with siRNA against human *SFMBT2* (M-026395-01-0005, GE Dharmacon) or control siRNA (sc-37007, Santa Cruz Biotechnology, USA). The efficiency of specific gene knockdown was confirmed with quantitative PCR. Transfection was performed with Lipofectamine 2000 (Invitrogen) according to the manufacturer's instructions. For stable transfection, LNCaP cells were transfected with GIPZ lentiviral SFMBT2 shRNA containing GFP (RHS4531, GE Dharmacon). GIPZ lentiviral control shRNA (RHS4346, GE Dharmacon) was used as a negative control. Stable cell lines were established by culturing LNCaP cells in media containing 1 μg/ml puromycin. Total 17 clones were obtained. Among them, a cell line showing lowest expression of SFMBT2 was selected by RT-PCR for the further study.

### Cell migration and invasion assay

Cell migration assays were performed using modified Boyden Chambers (Transwell, Corning Costar). LNCaP and VCaP cells were starved in serum free medium for 24hr, and then plated into the upper chamber of 24-well Transwell plate with 8μm pore size. Cells were incubated for 24 hr, the migrated cells were fixed, stained with cresyl violet, and counted. For invasion assays, cells were allowed to invade through a matrigel-coated membrane (BD Biosciences). After cells were plated into Transwell plate for 48 hr, cells on the upper side of the membrane were removed and the migrated or invaded cells were fixed, stained with cresyl violet, and counted.

### Chromatin immunoprecipitation (ChIP)

ChIP was performed as described previously [24]. Antibodies are described in Supplementary Table S3. Normal IgG (sc-2017, Santa Cruz Biotechnology) or normal IgM (sc-3881, Santa Cruz Biotechnology) was used as a negative control. Quantitative PCR was performed using the primers listed in Supplementary Table S4. The relative proportions of immunoprecipitated fragments were determined using the ΔCt comparative method based on the threshold cycle (Ct) value for each PCR reaction and normalized to input genomic DNA.

### Immunohistochemical analysis of SFMBT2 in prostate cancer tissues

Immunohistochemical analysis of prostate cancer tissue arrays (2 mm core diameter) was performed using the Leica BOND MAX autostainer (Leica Biosystems) at the Super Bio Chip Laboratories (Korea). Briefly, slides were deparaffinized at 60°C for 1 hour and then treated with Bond Dewax solution for 3 min at 72°C. Antigen retrieval was performed using Epitomic retrieval solution 2 (pH 9.0) for 20 min at 100°C. After endogenous peroxidases were blocked by incubation with hydrogen peroxide for 5 min, the tissue sections were incubated for 30 min at RT with anti-SFMBT2 antibody (1:150, 730036, Novex). Subsequently, tissue sections were incubated with a polymer-conjugated secondary antibody using the Leica Bond Polymer detection kit. The antigen was visualized with 3, 3′-diaminobenzidine (DAB) solution. The nuclei were counterstained with hematoxylin. The entire fields of tissues array were scanned with Leica BOND-MAX automated imaging system. Intensity values of images from whole tissue were analyzed with ImageJ software (NIH). According to previous report [72], the images were scored based on signal intensity (no staining = 0, weak staining = 1, moderate staining = 2, strong staining = 3) and the extent of stained cells (0% = 0, 1~10% = 1, 11~50% = 2, 51~80% = 3, 81~100% = 4). Scoring was done by two reviewers, who blind to the results. The final score was determined by multiplying the intensity scores with the extent of scores of stained cells. The final scores are in range from 0 to 12. If the score is 0 to 4, it's called “SFMBT2 low”. If the score is 5 to 8, it's called “SFMBT2 moderate”. A score of 8~12 is called “SFMBT2 high”. Fisher's exact test was used to calculate P values.

### *In vivo* metastasis assay

*In vivo* metastasis assay were performed using mouse tail vein or intraprostatic injection. The committee for experimental animal research at Sogang University approved the animal experiments. Male athymic BALB/c nude mice (5 weeks old, 21 g of average body weight; DBL, Korea) were used for *in vivo* metastasis experiment with two biological repeats (n=3/group). For tail vein injection, one million LNCaP cells stably transfected with shContol-GFP or shSFMBT2-GFP in 200 μl PBS were injected into tail vein. The mice were imaged for fluorescence using FOBI system (NEO Science). At 15 weeks after injection, mice were dissected and organs were removed and photographed. For intraprostatic injection, male athymic BALB/c nude mice were anesthetized with intraperitoneal injection of 2, 2, 2-tribromoethanol (0.24 μg/g of body weight; Sigma Aldrich) and then placed in a supine position [73]. A midline incision was made in the lower abdomen and the prostate was exteriorized. One million LNCaP cells stably transfected with shContol-GFP or shSFMBT2-GFP in 50 μl PBS were injected to the dorsolateral side of the prostate. The incision was closed with sutures. At 5 weeks after injection, mice were dissected and organs were removed and photographed.

## SUPPLEMENTARY MATERIALS FIGURES AND TABLES


