# Supplementary material for: SFMBT2 (Scm-like with four mbt domains 2) negatively regulates cell migration and invasion in prostate cancer cells
Source: Oncotarget. 2016 Jun 21;7(30):48250–64. doi: 10.18632/oncotarget.10198 (PMC5217015; doi:10.18632/oncotarget.10198)
Supplement: Supplementary file 1 [file oncotarget-07-48250-s001.pdf]

SFMBT2 (Scm-like with four mbt domains 2) negatively regulates cell migration and invasion in prostate cancer cells

SUPPLEMENTARY MATERIALS AND METHODS

Cell culture

RWPE-1, LNCaP, VCaP and PC3 cells were obtained from the American Type Culture Collection and DU145 cells were purchased from Korean Cell Line Bank. RWPE-1 cells were maintained in Keratinocyte-SFM (Invitrogen) supplemented with 10% fetal bovine serum, 0.05 mg/ml BPE, 5 ng/ml EGF, and antibiotics. LNCaP and DU145 cells were cultured in RPMI 1640 Medium supplemented with 10% fetal bovine serum and antibiotics. VCaP and PC3 cells were maintained in DMEM with 10% fetal bovine serum and antibiotics.

RNA extraction and quantitative PCR

After isolation of total RNA with TRIzol reagent (Ambion), 500 ng of total RNAs were reverse transcribed using the PrimeScript II 1st strand cDNA Synthesis Kit (Takara). The resulting cDNA was subjected to real-time PCR with SYBR Green I Mix (Takara) using Light Cycler (Roche). PCR condition used to amplify all genes were 30 s at 95°C and 40 cycles of 95°C for 5 s, 60°C for 34 s. Expression data were calculated from the cycle threshold (Ct) value using the ΔCt method for quantification. GAPDH mRNA level was as used for normalization. Oligonucleotide primers of real-time PCR are described in Supplementary Table S1.

Plasmid constructs and promoter reporter assay

Human gene promoters were amplified using oligonucleotide primers (Supplementary Table S2) and cloned into pGL3 vector (Promega). Mutation of YY1 binding site in MMP-9, MMP-26 and N-CoR gene promoters was performed by PCR using oligonucleotide (Supplementary Table S2). LNCaP cells were co-transfected with gene promoter-driven firefly luciferase and control thymidine kinase promoter-driven renilla luciferase. Cells were harvested at 48 hr after transfection and promoter reporter activity was determined using the Dual-Luciferase Assay System (Promega) with a Lumat BL 9507 luminometer (Berthold technologies). Firefly luciferase activity of gene promoter was normalized to *Renilla* luciferase activity.

Immunoprecipitation and western blot analysis

Immunoprecipitation and Western blotting were performed as described previously [24]. Antibodies are described in Supplementary Table S3. Normal IgG (sc-2017, Santa Cruz Biotechnology) or normal IgM (sc-3881, Santa Cruz Biotechnology) was used as a negative control. Western blots were analyzed quantitatively using the ImageJ software (NIH).

Zymography

Equal amount of protein was loaded and proteins were separated by SDS-PAGE in 10% and 12% gels polymerized in the presence of gelatin or casein, respectively. Gels were washed for 20min in 2.5% Triton X-100 and incubated at 37°C for 24~48 hr in renaturing buffer consisting of 50 mM Tris-HCl (pH 7.5), 150 mM NaCl and 10 mM $CaCl_2$. Gels were stained in 1% Coomassie blue and destained to visualize bands of gelatin or casein substrate digestion. Zymography was analyzed quantitatively using the ImageJ software (NIH).

Immunocytochemistry

Cells were fixed for 15 min with 4% paraformaldehyde in PBS and permeabilized with PBST solution (0.5% Triton X-100 in PBS) for 30 min. After blocking of cells with 5% BSA in PBST solution for 1hr, cells were incubated with the anti-NF-κB p65 antibody (1: 250, ab7970, Abcam) overnight at 4°C. Antigens were detected with the secondary antibodies conjugated to TR (Sigma-Aldrich). Nuclei were identified using DAPI staining. Images were acquired with a confocal microscope (Leica TCS SPE).

Histology

For histological analysis, organs were excised from mice at 5 or 15 weeks after injection. Organs were fixed with 4% paraformaldehyde solution and embedded in paraffin. The sections were deparaffinized by xylene and rehydrated. Hematoxylin and eosin (DAKO) staining was performed.

Statistical analyses

All quantitative data are presented as mean ± S.E.M. The differences between three groups were evaluated by a paired t-test.

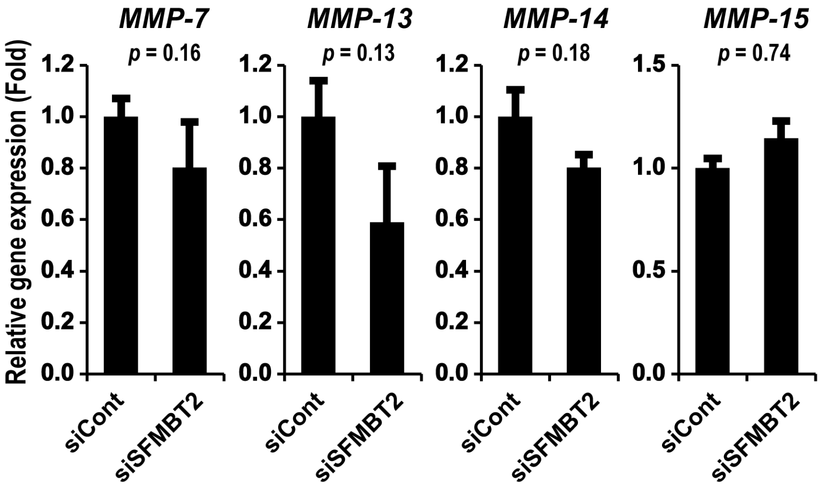

Supplementary Figure S1: Gene expression of MMP in SFMBT2 knockdown LNCaP cells. After control or SFMBT2 siRNA were transfected, LNCaP cells were subjected to RNA extraction (n=3). Transcripts of *MMP-7*, *MMP-13*, *MMP-14*, *MMP-15*, and *GAPDH* were determined by quantitative PCR. All data represent mean ± S.E.M.

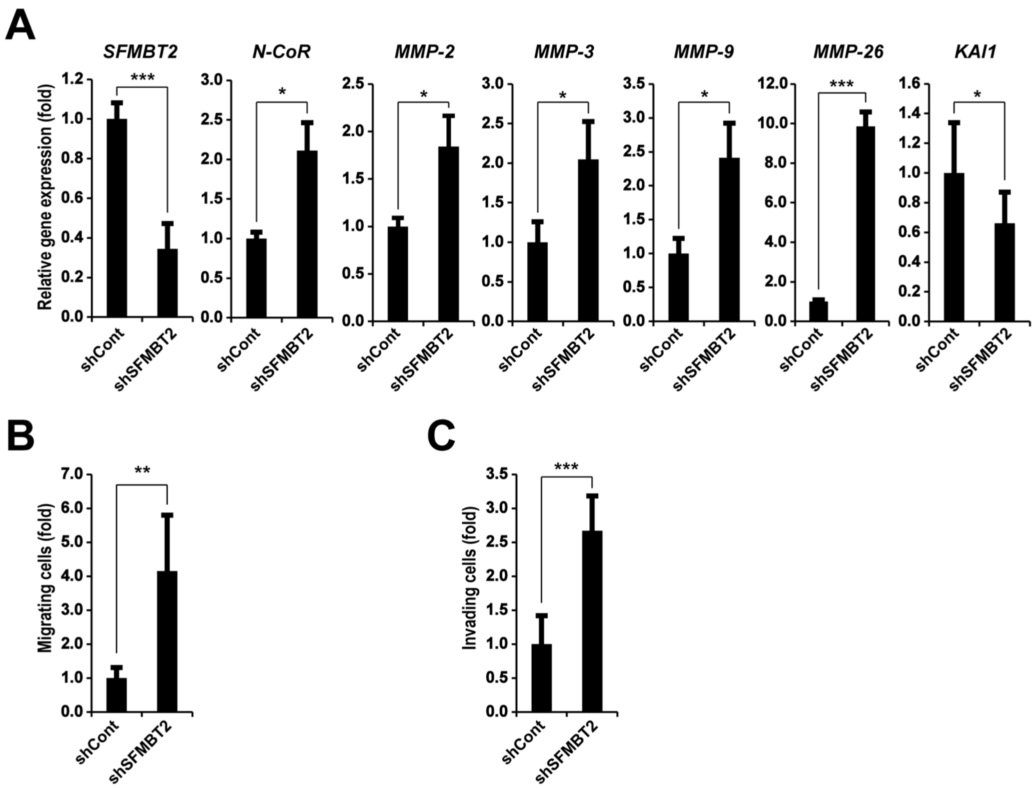

Supplementary Figure S2: SFMBT2-mediated cell migration and invasion in VCaP cells. A. After control or SFMBT2 shRNA were transfected, VCaP cells were subjected to RNA extraction (n=3). Transcripts of *SFMBT2*, *N-CoR, MMP-2, MMP-3, MMP-9, MMP-26, KAI1* and *GAPDH* were determined by quantitative PCR. **B.** After control or SFMBT2 shRNA were transfected, VCaP cells were subjected to a cell migration assay using a modified Boyden chamber containing uncoated Transwell polycarbonate membrane filters (n=3). The migrated cells stained with cresyl violet were counted. **C.** After control or SFMBT2 shRNA were transfected, VCaP cells were subjected to a cell invasion assay using a Biocoat Matrigel invasion chambers (n=3). Invading cells on the membrane stained with cresyl violet were counted. All data represent mean ± S.E.M.

**MMP-2 gene promoter (YY1)**	-850 -844 -671 -665 -504 -498<br>TTTG**CCATCTT**GGTT····GTTC**AAGATGG**AGTC····GTTT**CCATCTC**TCTC·<br>-490 -484 -425 -419 -391 -385<br>···CTTT**CCATCTC**TGTT····CTGA**CCATCTAT**CAT····CTGA**CCATTCC**TTCC
**MMP-3 gene promoter (YY1)**	-1192 -1186 -1149 -1143 -1035 -1025<br>AAGC**AAAATGG**GCAC····GTAT**CCATCAT**TCTA····TGAG**GAGATGGATGG**TGGC<br>-851 -843 -697 -691 -675 -662<br>····ATTT**AGGATGGA**TTCT····ACTA**TTTATGG**AGCA····GGGC**CCATCACCCATTAA**TAAA<br>-581 -575 -107 -101 -92 -86<br>····CTGC**CCATGAG**GTTT····TTTG**GAAATGG**TCCTGCTG**CCATTTG**GATG
**MMP-9 gene promoter (YY1)**	-628 -620 -522 -514 -63 -56<br>CCTG**CGGAAGACA**GGGG····AGGC**TGTCAGGGA**GGGA····TGCC**TGTCAAGG**AGGG
**MMP-26 gene promoter (YY1)**	-740 -732 -436 -428 -367 -359<br>CCTC**TGTCGCCCA**GGCT····ATTA**CAGCAGACA**TTAT····TGAA**TGTCTCTGA**CTGG
**N-CoR gene promoter (YY1)**	-760 -754 -722 -716 -224 -218<br>TTCT**CCATGTT**CGTC····TTAA**GTGATGG**CCCG····GGGC**ACGATGG**CAGC
**TAB2 gene promoter (YY1)**	-961 -953 -765 -759 -432 -424<br>CCCT**AGCAAGACA**GGCG····GCCG**CCATATT**GTGT····GAGG**TGTCCCCCC**TGCC
**p65 gene promoter (YY1)**	-965 -959 -538 -532 -474 -468<br>TTCT**CCATCTG**GAGA····CGTC**CCATCCG**GGCT····GCTC**CCATTCC**CCCA
**p50 gene promoter (YY1)**	-1152 -1146 -1113 -1107 -891 -885<br>GAGG**AGGATGG**AGAG····CATG**CCATAAG**CAAT····CCAG**TGAATGG**TGGC····<br>-782 -776 -770 -764 -562 -556 -512 -506<br>AAAA**CCATTGC**TACCA**CCATCAC**TATA····CGCT**TAAATGG**AATA····GGAT**CCATGCC**GACC<br>-473 -467 -403 -397<br>····AGGG**GCTATGG**ACCG····CCGA**CCATTGA**TTGG
**MMP-9 gene promoter (p65)**	-605 -596<br>CCAG**TGGAATTCCC**CAGC
**MMP-26 gene promoter (p65)**	-424 -415<br>ATTA**TTTATTTCCC**TTGA
**N-CoR gene promoter (p65)**	-612 -603 -110 -101<br>AACC**GTCGTTTCCC**CAGC····ACCC**GGGAAGCTCC**GCCG

Supplementary Figure S3: Identification of YY1 and NF-κB p65 binding sites in the gene promoters by *in silico* bioinformatic analysis.

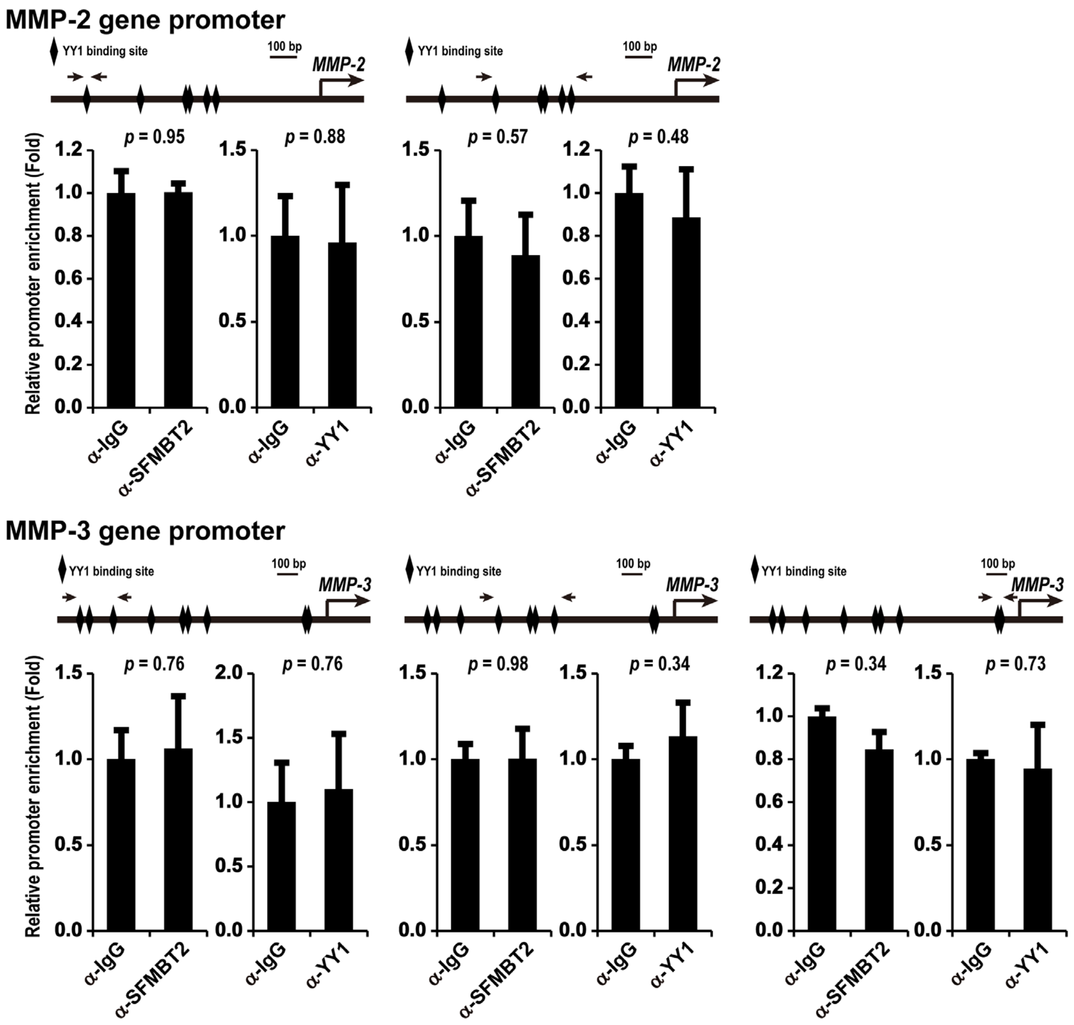

Supplementary Figure S4: SFMBT2 and YY1 are not enriched at the MMP-2 and MMP-3 gene promoters in LNCaP cells. A ChIP assay was performed in LNCaP cells using anti-SFMBT2 and anti-YY1 antibodies, respectively (n=3). The occupancy of each protein was determined by quantitative PCR in MMP-2 and MMP-3 gene promoters encompassing the putative YY1 binding sites using oligonucleotide primers (arrows). ChIP using normal IgG was performed as a negative control. All data represent mean ± S.E.M.

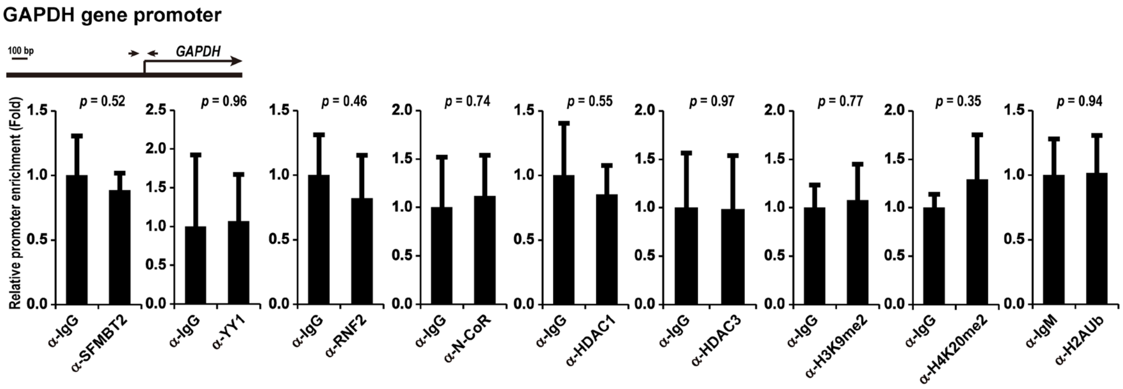

Supplementary Figure S5: No significant enrichment of SFMBT2, YY1, RNF2, N-CoR, HDAC1, HDAC3, H2AK119Ub, H3K9me2, and H4K20me2 on the GAPDH gene promoter in LNCaP cells. A ChIP assay was performed in LNCaP cells using anti-SFMBT2, anti-YY1, anti-RNF2, anti-N-CoR, anti-HDAC1, anti-HDAC3, anti-H2AK119Ub, anti-H3K9me2, and anti-H4K20me2 antibodies, respectively (n=3). The occupancy of each protein was determined by quantitative PCR in the GAPDH gene promoter. ChIP using normal IgG or IgM was performed as a negative control (n=3). All data represent mean ± S.E.M.

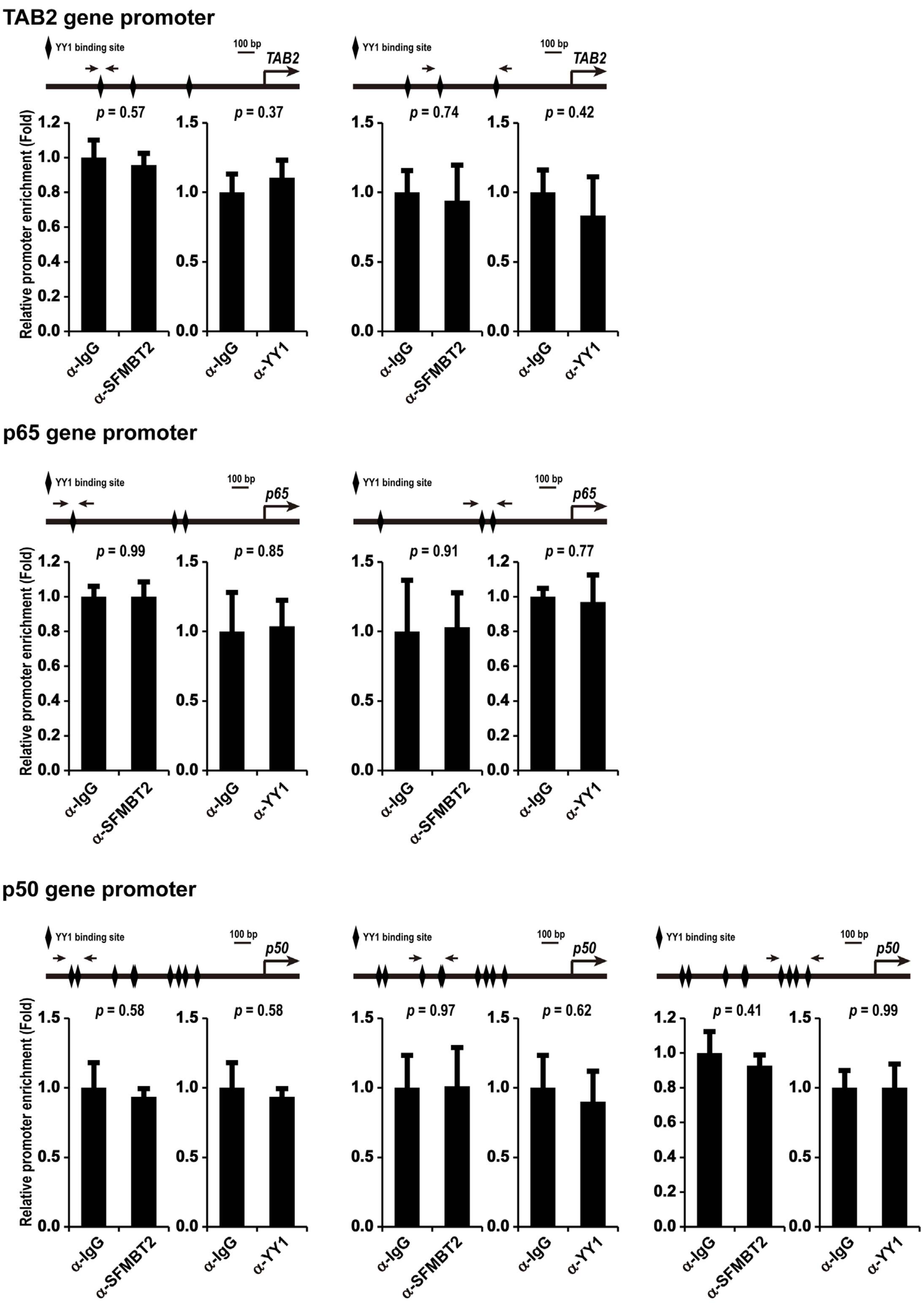

Supplementary Figure S6: SFMBT2 and YY1 are not enriched on the TAB2, p65, and p50 gene promoters in LNCaP cells. A ChIP assay was performed in LNCaP cells using anti-SFMBT2 and anti-YY1 antibodies, respectively (n=3). The occupancy of each protein was determined by quantitative PCR in the TAB2, p65, and p50 gene promoters encompassing the putative YY1 binding sites using oligonucleotide primers (arrows). ChIP using normal IgG was performed as a negative control. All data represent mean ± S.E.M.

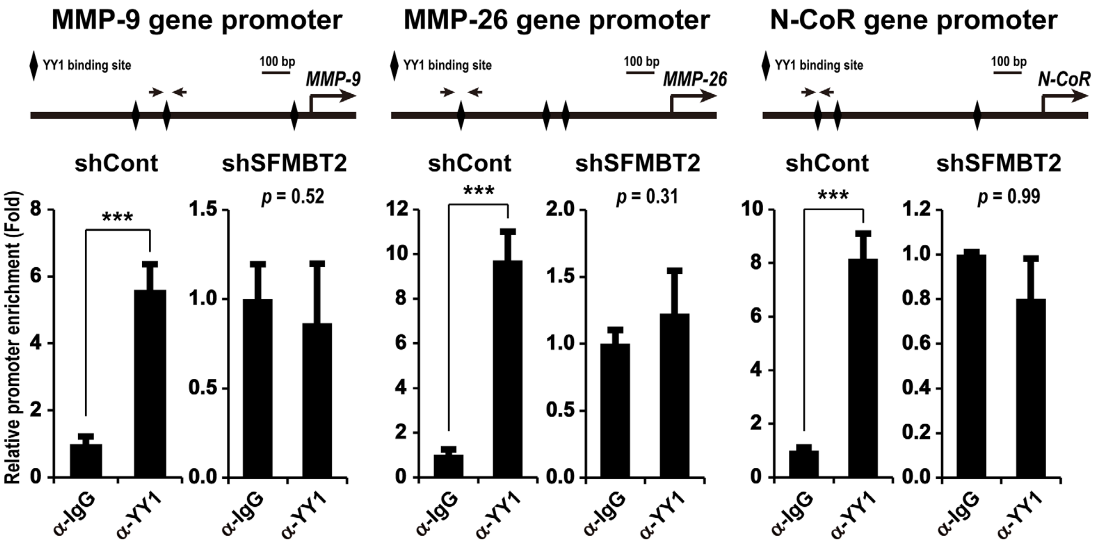

Supplementary Figure S7: YY1 is not enriched on the MMP-9, MMP-26, and N-CoR gene promoter in SFMBT2 knockdown LNCaP cells. After control (shCont) or SFMBT2 shRNA (shSFMBT2) were transfected stably in LNCaP cells, A ChIP assay was performed using the anti-YY1 antibody (n=3). The occupancy of each protein was determined by quantitative PCR in MMP-9, MMP-26, and N-CoR gene promoters encompassing the YY1 binding site. ChIP using normal IgG was performed as a negative control. All data represent mean ± S.E.M. Significance values was *** $P \leq 0.005$.

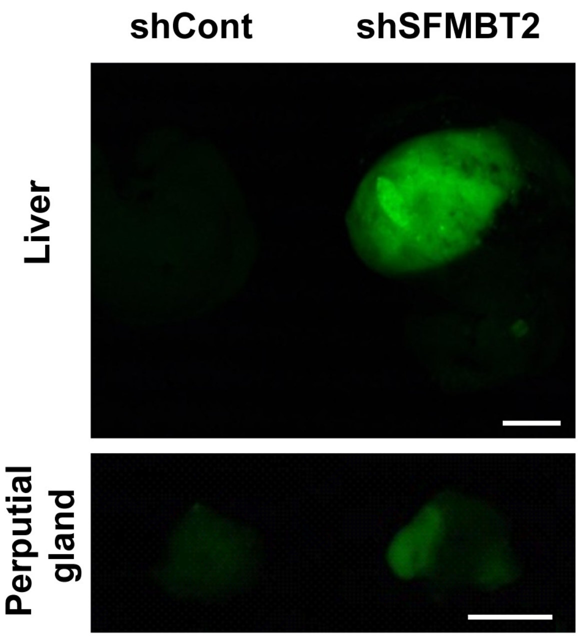

Supplementary Figure S8: *In vivo* imaging of liver and perputial gland tissue in (shCont)- or SFMBT2 shRNA (shSFMBT2)-GFP LNCaP cell-injected mice. LNCaP cells (1x10^6 cells) transfected stably with control (shCont)- or SFMBT2 shRNA (shSFMBT2)-GFP were injected into the tail vein of nude mice. Representative photomicrographs show fluorescence in dissected liver and perputial gland at week 15 post injection. Scale bar; 1 cm.

Supplementary Table S1: Oligonucleotide primers used for quantitative PCR in this study

gene	Forward primer	Reverse primer
SFMBT2	TGACGTAGTCATCGCGGATTT	ACCAGTCAAGTCACGTATGAGAA
MMP-2	TACAGGATCATTGGCTACACACC	GGTCACATCGCTCCAGACT
MMP-3	AGTCTTCCAATCCTACTGTTGCT	TCCCCGTCACCTCCAATCC
MMP-7	TGTATGGGGAACTGCTGACA	AGACTGCTACCATCCGTCCA
MMP-9	AGGACGGCAATGCTGATG	TCGTAGTTGGCGGTGGTG
MMP-13	ACTGAGAGGCTCCGAGAAATG	GAACCCCGCATCTTGGCTT
MMP-14	CGAGGTGCCCTATGCCTAC	CTCGGCAGAGTCAAAGTGG
MMP-15	GGCCGACATCATGGTACTCT	GAGGTTGTTTCCATGCAGGT
MMP-26	GGCCAGGTGGTATCTTAGGC	AGCTGACCAGTGTTCATTCTTG
KAI1	GGAAGCCCATGAGCATAGTG	GCCGACAAGAGCAGTTTCAT
N-CoR	GGAATCGAAGCGACCACGT	ACTAAAGGCAAAACCGCAGC
TAB2	CTCGAAGGCGCCTGAAAAGA	GGGTTTTGGTGGCACAGGAC
p65	TAGGAAAGGACTGCCGGGAT	CCGCTTCTTCACACACTGGA
p50	TGAGTCCTGCTCCTTCCAA	CTTCGGTGTAGCCCATTTGT
GAPDH	AGGTCGGTGTGAACGGATTTG	TGTAGACCATGTAGTTGAGGTCA

Supplementary Table S2: Oligonucleotide primers used for promoter cloning and mutation in this study

gene	Forward primer	Reverse primer
**Promoter cloning primer**		
N-CoR	AGGCTGGTCTCGAACTC	ACGACGGTTCCTCGTAAC
MMP-9	GGGGAGGATATCTGACCTGG	CTGCAGAGCTTGTGGGAACT
MMP-26	GTTTTTGAGACGGAGTCTCCCTC	CGCCTGTAGTCCCAGCTACTC
**Mutagenesis primer**		
N-CoR	AACTCCTGACCTTAAACAGCAACCCGCCT CGGCCTCCGGAGGCC	GGAGGCCGAGGCGGGTTGCTGTTTAAGGT CAGGAGTT
MMP-9	ACTGGAGGCTTTCAGACCAAAAAGCAAAG GATCCCTCCAGCTTCATC	GATGAAGCTGGAGGGATCCTTTGCTTTTTG GTCTGAAAGCCTCCAGT
MMP-26	TTTGAGACGGAGTCTCCCTCGTGATAAAC GGCTGGAGTGCAGTGGCGCG	CGCGCCACTGCACTCCAGCCGTTTATCAC GAGGGAGACTCCGTCTCAAA

Supplementary Table S3: Antibodies used in this study

Antibody	Cat #	Company	Application
SFMBT2	730036	Novex	WB, IP, ChIP
YY1	sc-7341	Santa Cruz Biotechnology	WB, ChIP
N-CoR	17-10260	Millipore	WB, ChIP
RNF2	ab3822	Abcam	WB, ChIP
H2AK119Ub	05-678	Millipore	ChIP
HDAC1	sc-7872	Santa Cruz Biotechnology	WB, ChIP
HDAC3	sc-11417	Santa Cruz Biotechnology	WB, ChIP
H3K9me2	ab1220	Abcam	ChIP
H4K20me2	ab9052	Abcam	ChIP
MMP-2	ab79781	Abcam	WB
MMP-3	sc-31074	Santa Cruz Biotechnology	WB
MMP-9	ab38898	Abcam	WB
MMP-26	sc-100558	Santa Cruz Biotechnology	WB
β-actin	LF-PA0209	Abfrontier	WB
HA	MMS101P	Covance	WB
p65	ab7970	Abcam	ChIP
p300	sc-7341	Santa Cruz Biotechnology	ChIP
AcH3	06-599	Millipore	ChIP
pIκB	ab7212	Abcam	WB
IkB	ab7217	Abcam	WB

WB : Western blot, ChIP : Chromatin immunoprecipitation, IP : immunoprecipitation

Supplementary Table S4: Oligonucleotide primers used for ChIP assay in this study

gene	Forward primer	Reverse primer
MMP-9 (YY1)	GGGGAGGATATCTGACCTGG	CTGCAGAGCTTGTGGGAACT
MMP-9 (p65)	GCCATGTCTGCTGTTTTCTAGAGG	CACACTCCAGGCCTCTGTCCTCTTT
MMP-26 (YY1)	GTTTTTGAGACGGAGTCTCCCTC	CGCCTGTAGTCCCAGCTACTC
MMP-26 (p65)	CACCACCACACCCGGCTAAT	AAGACCTGGCCTGGCACGAT
N-CoR (YY1)	AGGCTGGAGTGCAGTGG	GTCAGGAGTTCGAGACCAGC
N-CoR (p65)	CCTGGCTTTTCTAAAGGAACGTATGC	ATGCTGGGCGAGGCCGATTTG
MMP-2 site 1 (YY1)	TTTACTAGTTTAGGGGCTGAAGTCAG	ACAGTATGCAGTGAAGAAGCCAG
MMP-2 site 2 (YY1)	CAGCAGGTCTCAGCCTCATTT	TAGGACACCCTGCACTCCCT
MMP-3 site 1 (YY1)	AAGAAGAAAATGCAAAAGAGTTATAAAAA TGAGA	AACTTTTATAGTCCTCTTGCCACCT
MMP-3 site 2 (YY1)	AGGTGGCAAGAGGACTATAAAAGTT	TATTGACTGTCGTTGGATTTGCT
MMP-3 site 3 (YY1)	AAAGTAGGTTGTATCATCCTACTTTGAA	TTAAAGAGTGACAGTGTTTGTTTGGA
TAB2 site 1 (YY1)	GTCCTCCCTCCAGCCCTA	CAGCCCAGTCCAAATCAAAATAC
TAB2 site 2 (YY1)	GAGGGAGGGAGGGCTGA	TTACCCTTTGCCGCCGC
p65 site 1 (YY1)	TCTTCAGAGCCTGGGA	CACAGTGCAGGTAGCT
p65 site 2 (YY1)	TCGTAAGCTGGCGCTT	AATGTCCTACCCACCC
p50 site 1 (YY1)	AAACACTCCACCAAGAAGGTTTTTAT	ACTGTATTAGGTACAGGCTGGGGA
p50 site 2 (YY1)	AGCACTAAAGCAGCTCAGATGC	ACAAGGCATTGTTAGGCCTTTATG
p50 site 3 (YY1)	GATGTGAATGTAACTGAGACACGCT	GCTGGAGCCGGTAGGGAA
GAPDH	TACTAGCGGTTTTACGGGCG	TCGAACAGGAGGAGCAGAGAGCGA

Supplementary Table S5: Characteristics of prostate cancer patients in tissue array

Characteristics	
**Gender**	**Male**
**Mean age in year (range)**	**65.52 (44-85)**
**Mean preoperative PSA ng/ml (range)**	**27.26 (0.5-161)**
**pTNM pathlogic classfication**	**N (%)**
pT1	4 (7.55%)
pT2	13 (24.53%)
pT3	32 (60.37%)
pT4	4 (7.55%)
**Gleason score**	**N (%)**
4-7	23 (43.40%)
8-10	30 (56.60%)

N : Patient number, PSA : Prostate-specific antigen, pTNM : Pathological tumor-node-metastasis
pT2a : Tumor affects one-half of one lobe or less
pT2b : Tumor affects more than one-half of one lobe but not both lobes
pT3 : Tumor extends beyond the prostate

Supplementary Table S6: Expression level of SFMBT2 in normal and prostate cancer specimens that used in tissue array

	SFMBT2-low	SFMBT2-moderate	SFMBT2-high	Total
Normal prostate	1/8 (12.5%)	0/8 (0%)	7/8 (87.5%)	8
Prostate cancer	32/53 (60.4%)	17/53 (32.1%)	4/53 (7.5%)	53
Gleason score 4-7	8/22 (36.36%)	11/22 (50.00%)	3/22 (13.64%)	17
Gleason score 8-10	24/31 (77.42%)	6/31 (19.35%)	1/31 (3.23%)	22
